# Recent Advances of Rare-Earth Ion Doped Luminescent Nanomaterials in Perovskite Solar Cells

**DOI:** 10.3390/nano8010043

**Published:** 2018-01-15

**Authors:** Yu Qiao, Shuhan Li, Wenhui Liu, Meiqing Ran, Haifei Lu, Yingping Yang

**Affiliations:** School of Science, Wuhan University of Technology, Wuhan 430070, China; qiaoyu@whut.edu.cn (Y.Q.); 0121114430305@whut.edu.cn (S.L.); 204410@whut.edu.cn (W.L.); rmq@whut.edu.cn (M.R.); haifeilv@whut.edu.cn (H.L.)

**Keywords:** perovskite solar cells, rare-earth (RE) ion doped nanomaterials, upconversion, downconversion

## Abstract

Organic-inorganic lead halide based perovskite solar cells have received broad interest due to their merits of low fabrication cost, a low temperature solution process, and high energy conversion efficiencies. Rare-earth (RE) ion doped nanomaterials can be used in perovskite solar cells to expand the range of absorption spectra and improve the stability due to its upconversion and downconversion effect. This article reviews recent progress in using RE-ion-doped nanomaterials in mesoporous electrodes, perovskite active layers, and as an external function layer of perovskite solar cells. Finally, we discuss the challenges facing the effective use of RE-ion-doped nanomaterials in perovskite solar cells and present some prospects for future research.

## 1. Introduction

With the constant development of human civilization, energy shortage and environmental pollution have become more and more significant challenges in our world, and they are issues that need to be resolved urgently. Solar energy is an ideal alternative energy source as it is renewable, inexhaustible, and clean energy. Grätzel indicated that the supply of energy from the sun to the earth is gigantic, or about 10,000 times more than the global population currently consumes [1]. Therefore, solar energy is a valid way to overcome the challenge of energy shortages for human beings. Since the first introduction of perovskite materials into solar cells in 2009 by Kojima [2], perovskite solar cells (PSCs) have attracted a great amount of attention because of their easy preparation method, low cost, and high efficiency [2,3,4,5,6,7,8,9]. The power conversion efficiency (PCE) of PSCs had explosive growth from 3.81% to 22.1% in the past eight years [2,3]. Stefaan discovered in 2014 that the perovskite thin film has a high absorption coefficient with a particularly sharp onset and no apparent presence of deep states [4]. Hence, the issue surrounding a lack of efficiently absorbing solar energy in conventional solid-state thin film solar cells has been successfully settled in PSCs. In 2012, Snaith and co-workers reported success with insulated Al_2_O_3_ nanoparticles to replace the semi-conductive TiO_2_ nanoparticles and the first utilization of mixed-halide CH_3_NH_3_PbI_3−*x*_Cl*_x_* as a perovskite layer. This change boosted efficiency to 10.9%. It also demonstrated that the perovskite not only plays a role in sensitizers, but also can transport the electrons between different cell proportions [5]. In the same year, Etgar and colleagues reported discovering a hole-conductor-free mesoscopic perovskite/TiO_2_ heterojunction solar cell. They directly evaporated a gold film on the top of perovskite as the electrode and reported efficiency of 7.3%. This result indicated that the perovskite can also assume the role of a hole conductor [6].

Perovskite solar cells have been regarded as one of the most ideal alternatives to silicon solar cells because of their advantageous features such as their large light absorption coefficient, high charge carrier mobility, and high conversion efficiency. However, there are some critical challenges for improving photovoltaic performance and stability of PSCs especially regarding conversion efficiency. The perovskite sensitizer is limited by its bandgap (1.55 eV), which results in its absorption spectrum rising to 780 nm [7]. However, about 52% of the whole solar energy is in the near-infrared (NIR) region (λ > 700 nm). So, the energy loss of near-infrared (NIR) light led to the limit for the PCE of PSCs [10]. On the other hand, thermalization of charge carriers caused by absorbing high-energy photons from which energy is larger than the bandgap of the perovskite sensitizer also limits the performance of PSCs. Only one electron-hole pair matching the bandgap of the perovskite sensitizer is generated by absorbing a high-energy photon and the excess energy of the high-energy photon is transformed into heat, which is harmful to stability of PSCs [11]. Thus, understanding how to reduce thermalization loss is a key factor for the high performance of PSC devices.

In 1966, while working on Yb^3+^-Er^3+^ co-doped glasses for lasers, Auzel found a visible green emission arising from IR excitation [12]. Since then, rare-earth ion doped luminescent nanomaterials have attracted considerable attention. There are two common pathways for luminescence of rare-earth ion doped nanomaterials. One is the transition in RE ions involving the f^n^ configuration, known as the f–f transition. The other is the transfer of a 4f electron into the 5d sub-shell. The photoluminescence caused by an f–f transition has the characteristics of narrow emission peaks, small temperature quenching, some affect by the matrix, rich emission lines, and more. Meanwhile, the f–d transitions have sizeable intensities and their energies depend on the metal-ion surrounding environment [13]. Contributed by the unique optical properties of RE-ion-doped nanomaterials, they have been widely applied in lasers, bioimaging, and solar cells [14,15,16].

Besides the upconversion (UC) property of RE-ion-doped luminescent nanomaterials, which generate high-energy visible light after absorbing low-energy NIR light, downconversion (DC) emission of emitting two or more low-energy photons by taking up one high-energy photon may also be observed. Because of the specific band diagram of RE ions, both of the UC and DC luminescent nanomaterials have emission spectra in the range of 400–700 nm, which coincides with the absorption of PSC. Therefore, the application of rare-earth ion doped luminescent nanomaterials in PSC is beneficial for the efficiency improvement of PSC.

## 2. Application of Rare-Earth RE-Ion-Doped Upconversion (UC) Nanomaterials in Perovskite Solar Cells (PSC)

### 2.1. Structure of PSC

**Figure 1:**
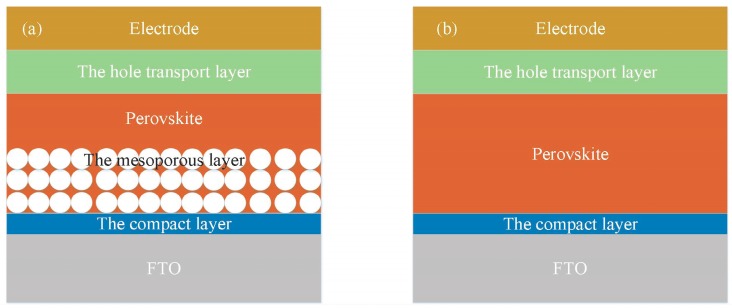
Structure of perovskite solar cells: (**a**) meso-porous structure; (**b**) planar pin junction structure.

There are two common types of PSC. These two types include mesoporous and planar structures, which have been depicted in Figure 1. As shown in Figure 1a, the compact layer has the ability to avoid direct contact between the fluorine-doped tin oxide (FTO) and hole transporting materials (HTM), which is also named the electron blocking layer. The inorganic semiconductor nanomaterials are commonly used to prepare the compact layer, such as TiO_2_, SnO_2_, ZnO, and so on. The mesoporous TiO_2_ film not only serves as an electron accepting and transporting layer (ETL) in PSCs, but also serves as a scaffold layer [6]. Meanwhile, the insulator oxide nanomaterials, such as Al_2_O_3_ and ZrO_2_, have also been investigated for creating the mesoporous film [5]. For example, the mesoporous Al_2_O_3_ film only served as a scaffold layer in PSC without the function of charge transportation between perovskite and Al_2_O_3_. Perovskite is used as a sensitizer to convert photons into electrons. Besides this, electron transfer and hole transfer can also occur in the perovskite layer. The HTM should be filled with the mesoporous layer to induce a heterojunction. The HTM is served as hole extracting from perovskite and can transfer the hole into the electrode. Because the PSC was approved for working without a mesoporous oxide layer, the planar pin junction structure with n-type thin TiO_2_, perovskite film, and p-type HTM film was constructed (as seen in Figure 1b).

### 2.2. Introduction of RE-Ion-Doped Upconversion Material

**Figure 2:**
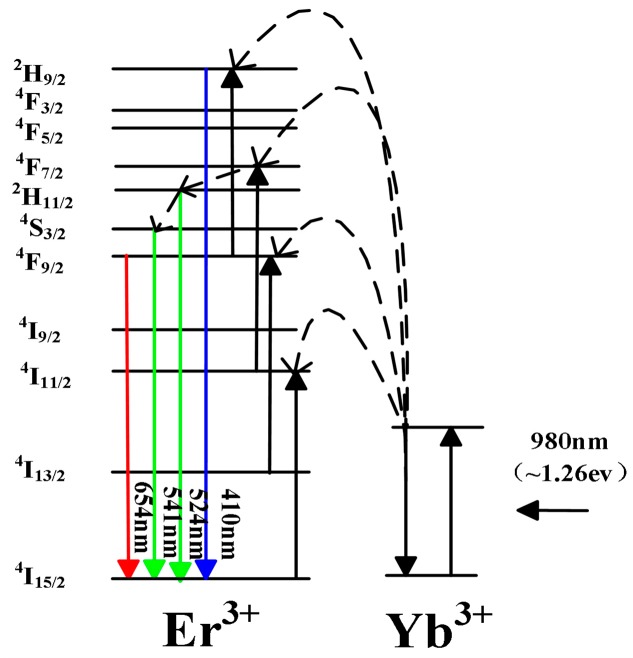
The detailed energy-level diagram and corresponding upconversion process of a hexagonal β-NaYF_4_:Yb^3+^, Er^3+^ nanoparticle.

Upconversion is a nonlinear optical phenomenon where the emission light has a shorter wavelength than the pump light source, which is an anti-Stokes process. Since the RE ions have long-lived excited states, the upconversion luminescent efficiency of RE-doped material is better than traditional photo-luminescent nanomaterials. Among the RE-doped upconversion nanomaterials, β-NaYF_4_:Yb^3+^, the Er^3+^ nanoparticle is the most efficient upconversion phosphor with bright green emission. This nanoparticle has attracted considerable attention [17,18,19]. As Figure 2 shows, under 980 nm laser excitation, the Yb^3+^ ion absorbs the photon energy and transfers it to the Er^3+^ ion for upconversion. Additionally, the wavelength of emission light is in the 410–654 nm range, which coincides with the absorption spectrum of PSC. Therefore, RE-doped upconversion material can be used to improve the PCE of PSCs, and RE ion doping TiO_2_ or perovskite nanocrystal is also an effective way for improving the photovoltaic performance of PSCs [20,21]. 

### 2.3. RE-Doped Upconversion Material in External Function Layer of PSCs

**Figure 3:**
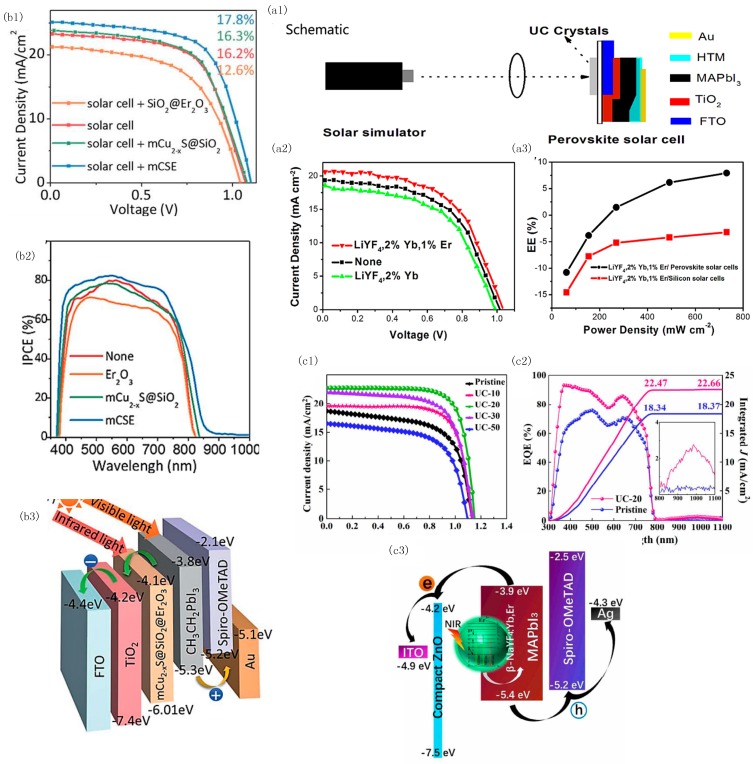
(**a1**) Schematic of the *J*–*V* characteristic curves measurement of the perovskite solar cells under the simulated sunlight excitation; (**a2**) *J*–*V* curves of the perovskite solar cells: LiYF_4_:2% Yb^3+^, 1% Er^3+^ crystals, LiYF_4_:2% Yb^3+^ crystals, and no crystal measured under simulated solar illumination with an intensity of 0.73 W·cm^−2^; (**a3**) Enhancement efficiencies (EE) of the perovskite solar cells and silicon solar cells with LiYF_4_:2% Yb^3+^, 1% Er^3+^ crystals dependent on the excitation power density. Reproduced with permission from [22]. American Chemical Society, 2016; (**b1**) *J*–*V* curves of the best-performing PSC, solar cell with SiO_2_@Er_2_O_3_, solar cell with mCu_2−*x*_S@SiO_2_, and solar cell with mCSE composites under simulated AM 1.5, 100 mW·cm^−2^ irradiance (simulated sunlight); (**b2**) IPCE curves of the best-performing PSC; (**b3**) Schematic illustration of the energy transfer process in CH_3_NH_3_PbI_3_ solar cells using mCSE composites. Reproduced with permission from [29]. Royal Society of Chemistry, 2017; (**c1**) *J*–*V* curves measured under AM 1.5 G of planar PSCs with different UCNCs additive amounts; (**c2**) IPCE spectra of UC-20 and pristine planar PSCs; inset is the magnification of 800–1100 nm region; (**c3**) Schematic energy transfer within a UCNCs-embedded planar PSC device. Reproduced with permission from [35]. Royal Society of Chemistry, 2017.

The fabrication of an UC layer on a PSC is a straightforward pathway to broaden the absorption of PSCs. In 2016, LiYF_4_:Yb^3+^, Er^3+^ upconversion single crystal was applied in PSCs as an independent luminescent upconverter by Chen et al. [22]. The photovoltaics response range of PSCs was 300–800 nm, which has a good match with the emission spectra of LiYF_4_:Yb^3+^, Er^3+^ upconversion NPs. The single crystal structure is beneficial for decreasing the defect states in LiYF_4_ and ensures transparency in the visible range [23]. As shown in Figure 3a1, the layer of LiYF_4_:Yb^3+^, Er^3+^ upconversion single crystal was placed in front of the PSCs. As compared to the PSC without LiYF_4_:Yb^3+^, Er^3+^ (11%), the PCE was enhanced by about 7.9% with LiYF_4_:Yb^3+^, Er^3+^ single crystal layer on PSC structure under the irradiation of simulated solar illumination (AM 1.5 G) with an intensity of 0.73 W·cm^−2^ (about seven or eight times that of solar power). Moreover, the researchers found that, when the intensity of simulated solar illumination is more than 0.2–0.73 W·cm^−2^, the utilization of LiYF_4_:Yb^3+^, Er^3+^ crystal in PSC is active for enhancing the efficiency of PSC.

### 2.4. RE-Doped Upconversion Material in ETL of PSCs

The introduction of RE-doped upconversion material into the commonly applied mesoporous electron transport layer (ETL) (such as m-TiO_2_) of PSC is the most popular way for using UC material in PSCs. The advantage of using UC material in PSCs is not only to broaden the absorption spectrum of PSC, which mainly depends on the UC process of RE-doped material, but also to improve the optical absorption of PSCs contributed by the scattering effects of the UC nanoparticles.

In 2016, M. He et al. reported the utilization of monodisperse NaYF_4_:Yb/Er upconversion nanoparticles (UCNPs) to replace the m-TiO_2_ as the mesoporous layer in PSCs for the first time [24]. By using the UCNPs in PSCs, the power conversion efficiency (PCE) was increased to 17.8%. Under the illumination of a 980 nm NIR laser with a power of 2 W, an efficiency of 0.35% was reported for the PCS. According to their experiments, the UCNPs intermediate distance and the thickness of UCNPs mesoporous layer have great effect on the performance of PSCs. When the average pore size of the mesostructured film composed of UCNPs was increased to 30.5 nm, the PCE was increased in size from 10.5% to 14.2%. A large-sized inter-nanoparticle spacing is expected to facilitate the penetration of perovskites inside the pores [25], which is useful for absorbing incidental photons and transporting charge carriers throughout the mesoporous electrode. A high PCE of 17.2% was recorded when the thickness of the UCNPs mesoporous layer was about 150 nm. Because of the insulating nature of NaYF_4_:Yb/Er nanoparticles, the thickness of UCNPs mesoporous layer exceeding 150 nm led to the reduction in *J*_sc_, *V*_oc_, and PCE. (*J*_sc_ is the short circuit current density and *V*_oc_ means the open circuit voltage of the solar cells).

In the same year, J. Roh et al. developed and reported on the NaYF_4_:Yb^3+^, Er^3+^ hexagonal nanoprisms (NYF) mixed with m-TiO_2_ as an upconverting mesoporous layer, which can absorb the incident NIR light and convert it into visible light that can be used in PSCs [26]. The best PCE was reached at 15.98% at a TiO_2_ mesoporous layer with 75 wt % NYF. This was a 13.47% enhancement in PCE compared with NYF free devices. The enhancement of PSC performance was attributed to (a) the larger number of carriers generated in perovskite via NIR photon upconversion of NYF, and (b) the slight improvement of charge migration in PSCs due to the electrons excited in perovskite having a high chance to be transferred through high crystalline NYF rather than through the TiO_2_ layer.

In 2016, M. Que et al. proposed the use of core-shell β-NaYF_4_:Yb^3+^, Tm^3+^/NaYF_4_ nanoparticles (NYF NPs) which had a high luminous intensity at visible range when incorporated into the m-TiO_2_ ETL in PSCs [27]. Optimal performance was recorded to be 16.9% when the NYF/TiO_2_ ratio was 0.25, which is about 20% enhancement when compared to the NYF-free PSCs. For better understanding of the mechanism behind using NYF in PSC for efficiency enhancement, the authors analyzed the field distribution from NYF NPs in PSCs with FDTD simulation. The FDTD simulation results indicated that the UC effect of NYF NPs and the light scattering by NYF NPs are both advantageous for enhancing PCE in PSCs.

The major challenge of using RE-doped upconversion material in solar cells is that the defect and ligands on the surface of RE-doped upconversion material would trap a large part of photo-generated electrons and decrease the photocurrent in solar cells. To overcome this issue, J. Hu et al. proposed a new configuration of UC material involving insulating SiO_2_ shell on the outside and positioning an internal β-NaYF_4_:Yb^3+^, Er^3+^ (NYEY) UC core to enhance the response of PSCs [28]. The β-NaYF_4_:Yb^3+^, Er^3+^@SiO_2_ (NYEY@S) nanoparticles were dispersed into the m-TiO_2_ ETL with a specific ratio of 0.5. The recorded PCE of NYEY@S incorporated PSC was 9.92%, which had an enhancement of 21% compared to the NYEY@S free devices and about 97% compared to the NYEY incorporated PSC.

In 2017, D. Zhou et al. developed a lanthanide oxides Er_2_O_3_ by additionally incorporating heavily doped semiconductor material Cu_2−*x*_S nanoparticles, which displayed an increase in UC efficiency (14.3% under 980 nm excitation) and also broadened the emission spectra and UC excitation spectra [29]. The mCu_2−*x*_S@SiO_2_@Er_2_O_3_ (mCSE) nanocomposites had a lower threshold power and a broader absorption range compared to normal UC material due to the localized surface plasmon resonance (LSPR) of mCu_2−*x*_S nanoparticles. The authors dispersed mCSE nanocomposites in the m-TiO_2_ ETL with a concentration range of 0–10 wt %. The recorded PCE was 17.8% at an optimal mCSE/m-TiO_2_ ratio of 0.5 wt %, which was 10% higher than the mCSE-free devices (16.2%). To better demonstrate the improved performance of PCE, the incident photon-to-current conversion efficiency (IPCE) spectra with mSCE demonstrated the broader generation of the photocurrent range (370~1000 nm) than the devices without mSCE (370~820 nm). As shown in Figure 3b3, the mCSE composites possessed an oxygen defect level about −4.1 eV, which matched well with the conduction band of m-TiO_2_ (about −4.2 eV). This indicated that the mCSE nanocomposites absorbed the NIR light, generated the photo-generated electrons, and then transferred the electrons to the conduction band of m-TiO_2_ to increase the photocurrent of PSCs.

This shows the effective progress in advancing the photoelectric properties of TiO_2_ by the RE ion doped procedure. In 2006, W. Wang et al. prepared Er^3+^-doped TiO_2_ with sol-gel method using tetrabutyl titanate as a precursor [30]. Under 980 nm laser excitation, three intense UC emission lines at 525, 550, and 660 nm were observed. The authors conducted the studies of the UC process. Y. Fan et al. prepared the Ho^3+^/Yb^3+^ co-doped TiO_2_ through sol-gel method and when the concentration of Yb^3+^ was 2.5 mol%, the intensity of fluorescence spectroscopy 543 nm. Conversion was the strongest under 980 nm excitation [31]. For PSCs, W. Li et al. [20] reported for the first time the application of Er and Yb co-doped TiO_2_(Er-Yb:TiO_2_) nanorod arrays as ETL. The PCE of PSC based on the Er-Yb:TiO_2_ presented an enhancement of 20.8% compared to the Er-Yb:TiO_2_ free devices.

### 2.5. RE-Doped Upconversion Nanomaterial in Active Layer of PSCs

The introduction of RE-doped upconversion nanomaterial into the commonly applied active layer of PSC, such as CH_3_NH_3_PbI_3_, was the most efficient way for improving photoelectric performance of PSCs. Unfortunately, the surfaces of most as prepared RE-doped upconversion nanomaterials usually have the long-chain alkyl molecules on the surface such as oleic acid (OA) and oleylamine (OM) [32], which limit the compatibility between RE-doped upconversion nanomaterials (RE-UCM) and perovskite precursors because the long-chain alkyl molecules create an insulating barrier around RE-UCM and impair the mixing ability of RE-UCM and the perovskite precursor. To address this drawback, a ligand-exchange strategy was developed to replace the originally capped long-chain alkyl ligands with specifically designed species, which makes the phase transfer of RE-UCM from a nonpolar solvent (e.g., n-hexane) to various polar media (e.g., DMF) [33].

**Scheme 1:**
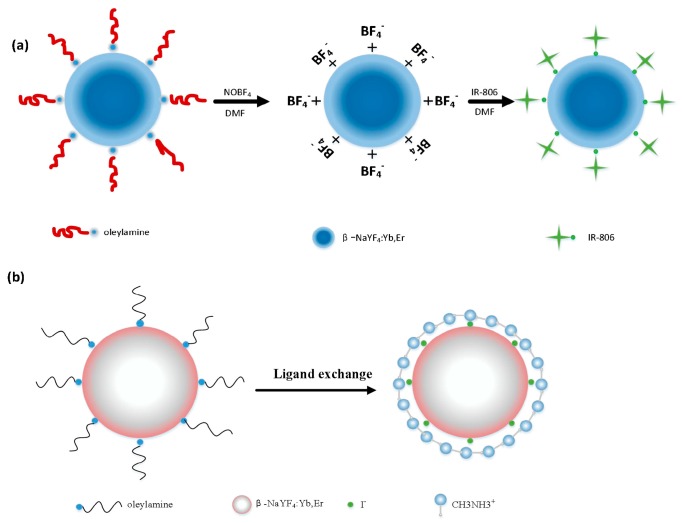
(**a**) Schematic preparation of hydrophilic IR806-UCNCs via a two-step ligand-exchange process. Reproduced with permission from [34]. Elsevier B.V, 2017; (**b**) Schematic preparation of UCNCs-embedded perovskite film via a ligand-exchange process. Reproduced with permission from [35]. Royal Society of Chemistry, 2017.

In 2017, a hydrophilic IR806 dye-sensitized upconversion nanocrystal IR806-β-NaYF_4_:Yb, Er (IR806-UCNCs) was fabricated by a two-step ligand-exchange approach to replace the OAm ligands on the surface of UCNCs with BF^4−^ and improve the stability of IR806-UCNCs in DMF (see Scheme 1a). It was proposed as a potential UC material for improving the response of PSCs [34]. By incorporating IR806-UCNCs into planar PSC, a PCE of 17.49% was recorded, which corresponded to 29% increment from that of IR806-UCNCs free devices. Moreover, a maximal PCEs of 0.382% was recorded under NIR irradiation (λ > 780 nm) using a solar light simulator with a 780 nm cutoff filter. During the same year, F. Meng et al. reported on the embedding of β-NaYF_4_:Yb, Er UCNCs into a CH_3_NH_3_PbI_3_ layer to fabricate NIR-enabled planar PSCs by using a ligand-exchange strategy for the first time [35]. As seen in Scheme 1b, methylammonium iodine (MAI) was used as a ligand to replace the OAm ligands capped on the surface of UCNCs in order to improve the compatibility between UCNCs and the perovskite precursor. Moreover, the MAI-capped UCNCs were used to grow the UCNC-embedded perovskite crystals. The advantage of the UCNC-embedded perovskite layer configuration was attributed to (a) a uniform pinhole-free morphology with enlarged crystal grains and enabled NIR absorption; and (b) the enhancement of energy transfer from UCNCs to perovskite. As seen in Figure 3c1, when the amount of UCNCs was 20 mg·mL^−1^, the superior performance of solar cells was recorded (*J*_sc_ = 19.60 mA·cm^−2^, *V*_oc_ = 1.13 V, FF = 72.98%), and the PCE was enhanced from 12.79 to 19.70% compared to its counterpart without UCNCs. Under 980 nm laser with an intensity of 3 W·cm^−1^, a PCE of 0.37% was recorded. Besides the ligand-exchange strategy, Laura et al. proposed the use of cucurbit [7] uril (CB [7]) to anchor the CH_3_NH_3_PbBr_3_ perovskite nanoparticles (PK) closely to NaYF_4_:Yb^3+^, Tm^3+^ upconversion nanoparticles (UC_n_), which lead to the development of UC_n_@PK_CB_ nanohybrids [21]. The authors corroborated the effective energy transfer from UC_n_ to PK under NIR light excitation by analyzing the emission spectra at λ_exc_ = 975 nm of UC_n_ and UC_n_@PK_CB_.

### 2.6. Application of RE-Doped DC Nanomaterials in PSC

**Figure 4:**
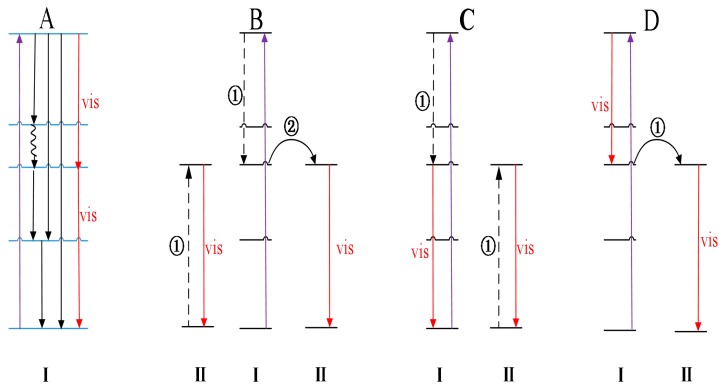
The schematic diagram of DC luminescence (① cross-relaxation ② energy transfer). (**A**) DC on a single ion by emission of two visible photons; (**B**–**D**) The possibility of DC on two ions. Reproduced with permission from [37]. The American Association for the Advancement of Science, 1999.

The downconversion (also called quantum cutting) process demonstrated that RE ions can result in two or more low-energy photons for absorbing one high-energy incident photon [36]. There are two types of RE ions (I and II) for the conceptions of DC (in Figure 4). As shown in Figure 4A, a single RE ion for DC luminescence is theoretically possible, but the competition of IR and ultraviolet (UV) prevent efficient DC luminescence on one RE ion. The use of two different RE ions called I and II can eliminate the losses in the IR and UV light by energy transfer between RE ions I and II. There are three possible DC mechanisms (Figure 4B–D) and two kinds of energy transfer between RE ions. Cross-relaxation (CR) usually refers to the energy transfer between identical ions (①) and energy transfer (ET) means resonant radiative transfer, resonant non-radiative transfer, and phonon-assisted energy transfer between different ions [37]. Figure 4B shows CR from ion I to II (①) and ET from ion I to II (②) leading to two-photon emission from ion II. Figure 4C,D indicate emission of photons from ion I and II by CR and ET mechanisms. For all three DC mechanisms, the theoretical visible quantum efficiency of 200% can be generated. In addition, the common RE ions (such as Te^3+^, Ce^3+^, and Lu^2+^) have a strong absorption at the ultraviolet range. It is beneficial for RE ions to excite at the ultraviolet range and accomplish DC luminescence. Consequently, the application of RE-doped DC nanomaterials in PSC have been proposed for optimizing the stability of solar cells by reducing thermalization losses.

In 2002, T. Trupke et al. proposed the use of DC layers placed in front of the silicon solar cells. The researchers presented a theoretical maximum conversion efficiency of 38.6% according to a detailed balance model for a solar cell system [11]. A significant improvement was achieved after comparing the device with DC layers placed in front of the silicon solar cells with a conventional solar cell (30.9–38.6%). This result indicates that the PCE of solar cells can improve by using DC layers.

For application of RE-doped DC nanomaterials in PSC, Wenhan Chen et al. prepared the uniform and efficient CeO_2_:Eu^3+^ nanophosphors by hydrothermal method and proposed it in 2017 as a potential DC material to improve the performance and light stability of PSCs [38]. The authors introduced the CeO_2_:Eu^3+^ nanophosphors into m-TiO_2_ ETL of PSC and an energy conversion efficiency of 10.8% was recorded, which had an increase of 35% compared to the referenced energy conversion efficiency without CeO_2_:Eu^3+^ nanophosphors. In addition, the PSC added with CeO_2_:Eu^3+^ nanocrystals revealed better stability under UV illumination compared with the CeO_2_:Eu^3+^ free devices. In the same year, Ling Jiang et al. developed and reported a transparent luminescent downconverting layer (LDL) by using Eu-4,7-diphenyl-1,10-phenanthroline (Eu-complex) to enhance the response of PSCs. The LDL was coated on the back of a FTO glass of PSCs [39]. When the concentration of Eu-complex was 1.5%, the PSCs coated with LDL reached a PCE of 15.44%, which was 17.5% higher than the uncoated PSCs. The UV-light-soaking measurement shows that the PSCs without LDL had a loss of above 50% of *J*_sc_ after UV irradiation for 10 h while the *J*_sc_ of the PSCs coated with LDL only degrading to 74% at the same condition. These measurements indicated that damage to the structure of perovskite solar cell by coating with LDL on the back could be alleviated after prolonged absorption of UV light and improved stability of PSCs.

## 3. Conclusions

Table 1 summary the published studies investigating the application of RE-doped nanomaterials in PSC. In conclusion, the application of RE-doped nanomaterials in PSCs is an elegant and useful strategy for improving the photoelectric properties and stability of PSCs due to the UC and DC luminescence of RE-doped nanomaterials. An extended absorption range to the NIR region and higher absorption of visible light can be expected after introducing RE-doped nanomaterials into the mesoporous ETL of PSCs because of the optical characterization (UC, DC process) and scattering effect of the RE-doped nanomaterials. Using RE-doped building blocks of ETL in PSCs can modulate their charge transportation properties. In addition, the external functional layer was created by using RE-doped nanomaterials on the back of FTO glass of PSCs to develop NIR solar radiation. Furthermore, an efficient energy transfer from RE-doped nanomaterials to perovskite and uniform perovskite crystals with enlarged crystal grains was achieved by incorporating the RE-doped nanomaterials into perovskite.

Significant improvement in experimental observation has been reported for the application of RE-doped nanomaterials in PSCs. However, there are still many issues for applying RE-doped nanomaterials in PSCs that need to be addressed. One important challenge is the low efficiency of existing RE-doped nanomaterials. Until now, there is no significant luminescence of RE-doped nanomaterials due to UC or DC under natural solar irradiation (AM 1.5). Additionally, the energy transfer mechanism between RE-doped nanomaterials and perovskite or m-TiO_2_ in PSCs has not been clearly illuminated since M. He et al. [22] introduced the RE-doped nanomaterials into PSCs for the first time. So we believe further studies regarding the energy transfer mechanism between RE-doped nanomaterials and perovskite or m-TiO_2_ in PSCs are important to boost the performance of PSCs. In addition, the major mechanism (scattering or photoluminescence) regarding performance enhancement in PSCs with RE-doped nanomaterials is not clear and more research will be necessary. Meanwhile, optimizing the device for introducing the RE-doped nanomaterials into PSCs is also an efficient way to develop high-performance PSCs. Based on the improvement in study of PSCs with RE-doped nanomaterials, the efficiency of PSCs will need further research.

## Figures and Tables

**Table 1:** Summary of the devices of application of RE-doped nanomaterials in PSC.

RE-Doped Nanomaterials	Device Structure	Application Mode	PCE (%)
LiYF_4_:Yb^3+^, Er^3+^	LiYF_4_:Yb^3+^, Er^3+^/FTO/TiO_2_/MAPbI_3_/HTM/Au	As external function layer, at the reverse of the FTO glass of PSCs	11.87% [22]
NaYF_4_:Yb^3+^, Er^3+^	cmTiO_2_/NaYF_4_:Yb^3+^, Er^3+^/MAPbI_3_/HTM/Ag	As mesoporous layer	17.8% [24]
NaYF_4_:Yb^3+^, Er^3+^	cm TiO_2_/NaYF_4_:Yb^3+^, Er^3+^-mp TiO_2_/MAPbI_3_/HTM/Au	As mesoporous layer (mixed with m-TiO_2_)	15.98% [26]
β-NaYF_4_:Yb^3+^, Tm^3+^@NaYF_4_ (NYTY@N)	cm TiO_2_/NYTY@N-mp TiO_2_/MAPbI_3_/HTM/Ag	As mesoporous layer (mixed with m-TiO_2_)	16.9% [27]
β-NaYF_4_:Yb^3+^, Er^3+^@SiO_2_ (NYEY@S)	cm TiO_2_/NYEY@S-mp TiO_2_/MAPbI_3_/C	As mesoporous layer (mixed with m-TiO_2_)	9.92% [28]
mCu_2-x_S@SiO_2_@Er_2_O_3_ (mCSE)	cm TiO_2_/mCSE-mp TiO_2_/MAPbI_3_/HTM/Au	As mesoporous layer (mixed with m-TiO_2_)	17.8% [29]
TiO_2_:Er^3+^, Yb^3+^	cm TiO_2_/TiO_2_:Er^3+^, Yb^3+^NRs/MAPbI_3−x_Cl_x_/HTM/Au	As mesoporous layer	13.4% [20]
IR806-NaYF_4_:Yb, Er	cm ZnO/IR806-NaYF_4_:Yb, Er^+^MAPbI_3_/HTM/Ag	Coupled with perovskite by ligand-exchange strategy	17.49% [34]
β-NaYF_4_:Yb, Er	cm ZnO/NaYF_4_:Yb, Er-MAPbI_3_/HTM/Ag	Coupled with perovskite by ligand-exchange strategy	19.70% [35]
CeO_2_:Eu^3+^	cm TiO_2_/CeO_2_:Eu^3+^-mp TiO_2_/MAPbI_3_/HTM/Ag	As mesoporous layer (mixed with m-TiO_2_)	10.8% [38]
Eu-complex LDL	Eu-complex LDL/FTO/cm TiO_2_/mp TiO_2_/MAPbI_3_/HTM/Au	As external function layer, at the reverse of the FTO glass of PSCs	15.44% [39]
